# Neuropsychological and neuroimaging characteristics of patients with mild cognitive impairment and negative-amyloid deposition

**DOI:** 10.3389/fneur.2025.1658712

**Published:** 2026-01-05

**Authors:** Xiangwei Dai, Junying Zhang, Xin Li, Yaojing Chen, Yanan Qiao, Shujuan Zhang, Kewei Chen, Lin Ai, Dantao Peng, Zhanjun Zhang

**Affiliations:** 1 Institute of Basic Research in Clinical Medicine, China Academy of Chinese Medical Sciences, Beijing, China; 2 Beijing Aging Brain Rejuvenation Initiative (BABRI) Centre, Beijing Normal University, Beijing, China; 3 State Key Laboratory of Cognitive Neuroscience and Learning, Beijing Normal University, Beijing, China; 4 Department of Neurology, China-Japan Friendship Hospital, Beijing, China; 5 Banner Alzheimer’s Institute, Phoenix, AZ, United States; 6 Department of Nuclear Medicine, Beijing Tiantan Hospital, Capital Medical University, Beijing, China; 7 Innovation Institute of Integrated Traditional Chinese and Western Medicine, Shandong First Medical University and Shandong Academy of Medical Sciences, Jinan, Shandong, China

**Keywords:** amyloid deposition, mild cognitive impairment, Alzheimer’s disease, gray matter atrophy, white matter disruption

## Abstract

**Objectives:**

Accumulating studies have reported that some mild cognitive impairment (MCI) patients without significant β-amyloid (Aβ) deposition on amyloid positron emission tomography (PET) can later develop Alzheimer’s disease (AD). Therefore, this study profiled the cognitive and neural characteristics of MCI patients with negative Aβ deposition to better understand potential features associated with an increased risk of AD progression.

**Methods:**

Thirty-seven MCI patients and 32 normal controls (NCs) underwent neuropsychological assessments, structural magnetic resonance imaging, and diffusion tensor imaging scans. MCI patients were stratified into amyloid-positive (Aβ_pos_; *n* = 18) and amyloid-negative (Aβ_neg_; *n* = 19) groups based on ^18^F-florbetapir PET. We compared cognitive performance, white matter (WM) integrity, and gray matter volume (GMV) across the three groups and further examined the interplay among brain structural alterations and cognitive changes.

**Results:**

Cognitively, relative to NCs, participants in Aβ_neg_ MCI group showed significant deficits in multiple cognitive domains including episodic memory, attention, and executive function, as those in Aβ_pos_ MCI group did. Both MCI subgroups exhibited extensive disruptions of WM integrity. Direct comparisons between the Aβ_neg_ and Aβ_pos_ groups revealed that Aβ-related structural changes were predominantly localized to the left hippocampus and adjacent regions. The increased Aβ deposition was closely associated with elevated mean diffusivity in the left hippocampal portion of the cingulum and reduced GMV of the left hippocampus. Moreover, the GMV of hippocampus could mediate the impact of WM disruption on episodic memory performance.

**Conclusion:**

Aβ_neg_ MCI patients who exhibit AD-like cognitive and structural abnormalities, particularly involving the hippocampus, may be associated with advanced cognitive decline or dementia progression. These results may help identify high-risk individuals within the heterogeneous Aβ_neg_ MCI population.

## 1 Introduction

The abnormal deposition of β-amyloid (Aβ) proteins is a hallmark pathology feature of Alzheimer’s disease (AD), beginning early in the AD continuum (1, 2). It is well established that Aβ deposition can initiate a cascade of downstream pathological events, including aberrant tau protein phosphorylation, neurofibrillary tangles, neuroinflammation, synaptic loss, neuronal death, and brain atrophy (3). However, Aβ accumulation often starts decades before the appearance of clinical symptoms, following a non-linear pattern (4, 5). This creates uncertainty regarding the onset of AD-related pathological progression and makes it challenging to investigate the early pathological alterations occurring during the initial stage of the disease.

Mild cognitive impairment (MCI) refers to a transitional stage between normal cognitive aging and dementia (6) and is generally regarded as a critical window for intervention. Currently, MCI patients with positive Aβ status (Aβ_pos_) are considered part of the AD continuum and are at risk for developing dementia (7). Compared with their Aβ-negative (Aβ_neg_) counterparts, Aβ_pos_ MCI patients exhibit greater neural changes, such as gray matter (GM) atrophy, cerebral hypometabolism, and abnormal brain functional activity, which are closely linked to faster cognitive decline and dementia progression (8–10).

Nevertheless, the absence of significant Aβ deposition does not preclude MCI patients from further cognitive decline or eventual dementia (11, 12). Although Aβ_neg_ MCI usually represents a heterogeneous population characterized by various non-AD pathophysiological processes, such as cerebrovascular injury, TDP-43 pathology, or age-related neurodegeneration (7, 13), it should be noted that 21% of individuals with Aβ_neg_ MCI progressed to AD within approximately 2.5 years (14), with an equivalent annual rate of approximately 9%, which exceeds that of cognitively healthy older adults. In addition, it is reported that both the rate of amyloid accumulation and the baseline levels of Aβ in patients with Aβ_neg_ MCI could predict early tau deposition in cortical Braak regions associated with AD (15). Moreover, some “AD-like” neural characteristics have also been reported among Aβ_neg_ MCI patients, such as hippocampal deformation and cortical thinning (9, 16). These findings suggested that there may be a close link between subthreshold Aβ and subsequent pathology progression. Since the biological processes underlying subthreshold Aβ remain unclear, more investigation into the cognitive and neural characteristics of Aβ_neg_ MCI patients is warranted.

In this study, we compared neuropsychological performance, white matter (WM) integrity, and GM volume between Aβ_neg_ and Aβ_pos_ MCI groups, while also including a group of normal controls (NCs), providing the same control group for each MCI subgroup. Based on that, we further examined the relations between Aβ-related brain structural changes and cognitive performance. These findings could help detect sensitive neural characteristics in Aβ_neg_ MCI patients that are related to the risk of dementia progression, thereby facilitating the identification of individuals at high risk for AD progression.

## 2 Materials and methods

### 2.1 Participants

The data for this study were obtained from the Beijing Aging Brain Rejuvenation Initiative (BABRI), a cohort study aimed to examine brain and cognitive changes during aging (17). The inclusion criteria for participants were as follows: (1) aged 50 years or older; (2) able to complete a battery of neuropsychological tests; and (3) availability for magnetic resonance imaging (MRI) data acquisition. The exclusion criteria included: (1) structural abnormalities other than cerebrovascular lesions, such as tumors, subdural hematomas, or contusions that could impair cognitive function; (2) history of addictions or psychiatric diseases (e.g., depression) or treatments that could affect cognitive function; (3) vessel diseases, such as cortical or subcortical infarcts; and (4) diseases involving white matter lesions (WMLs), such as multiple sclerosis. Additionally, patients diagnosed with vascular dementia or Lewy body dementia by neurologists were excluded.

The diagnosis of MCI was based on Petersen’s criteria (18), which included: (1) subjective memory complaints; (2) objective evidence of cognitive impairments (1.5 standard deviations (SD) below the age- and education-adjusted norm on one or more cognitive tests); (3) relatively preserved general cognitive function; and (4) maintained activities of daily living (score of zero on the Activities of Daily Living scale).

In total, 69 native Chinese participants were included in the present study, including 37 patients with MCI (19 Aβ_neg_ and 18 Aβ_pos_ MCI patients; see section 2.3. PET acquisition and preprocessing) and 32 NCs.

### 2.2 Neuropsychological assessments

All participants completed a set of neuropsychological tests to assess cognitive function. These tests were as follow: The general cognitive function was tested using the Chinese version of the Mini-Mental State Examination (MMSE) (19); memory was tested using the Auditory Verbal Learning Test (AVLT) (20), the Digit Span Test (DST; a subtest of the Wechsler Adult Intelligence Scale-Chinese revision), and the Rey-Osterrieth Complex Figure Test (ROCF) (21); executive function was tested using the Stroop Color-Word Test (SCWT) (22) and the Trail Making Test (TMT) (23); spatial processing was tested using the Clock Drawing Test (CDT) (24) and the ROCF-copy test (21); attention was tested using the Symbol Digit Modalities Test (SDMT) (25) and the TMT-A (23); language was tested using the Boston Naming Test (BNT) (26) and the Verbal Fluency Test (VFT) (27).

### 2.3 PET acquisition and preprocessing

A Discovery TM PET/CT Elite scanner (General Electric) was used in three-dimensional (3D) scanning mode, with 47 slices of 3.25-mm thickness, and covered the entire brain. The participants received an intravenous bolus of approximately 370 MBq (10 mCi) of 18F-florbetapir. A 10-min PET scan was acquired beginning 50 min post-injection. Two nuclear medicine physicians, who were trained to perform binary interpretation of florbetapir PET scans using an online training program offered by Eli Lilly, classified each MCI patient as Aβ_pos_ or Aβ_neg_.

For further quantitative analysis, the mean standard uptake value ratio (SUVr) was calculated. The SUVr was calculated following established procedures described in previous studies (28). In detail, all PET images were first normalized to the standard Montreal Neurological Institute (MNI) space using the SPM12 software package.^1^ Then, the whole cerebellum was selected as the reference region, and the florbetapir standardized uptake value (SUV) was measured from the frontal cortex, temporal cortex, precuneus, parietal cortex, anterior cingulate and posterior cingulate. The SUVr was obtained by dividing the mean SUV of the 6 regions of interest (ROIs) by the SUV of the reference region.

### 2.4 MRI data acquisition and preprocessing

MRI data were collected using a Siemens MAGNETOM Prisma 3T MRI system at the Imaging Center for Brain Research, Beijing Normal University. Participants were instructed to remain awake, relax with their eyes closed, and remain as motionless as possible. The T1-weighted structural images were acquired using 3D magnetization-prepared rapid gradient echo sequences: [192 sagittal slices, repetition time (TR) = 2,300 ms, echo time (TE) = 2.32 ms, slice thickness = 0.90 mm, flip angle = 8°, and field of view (FOV) = 240 mm × 240 mm]. DTI was acquired using a single-shot echoplanar imaging sequence [coverage of the whole brain, 2 mm slice thickness with no interslice gap, 75 axial slices, TR = 8,000 ms, TE = 60 ms, flip angle = 90°, 30 diffusion directions with b = 1,000 s/mm^2^, and an additional image without diffusion weighting (i.e., b = 0 s/mm^2^), and acquisition matrix = 128 × 128].

MATLAB R2012b (MathWorks, MA, United States) and SPM12 with default parameters were used to preprocess the structural images. The modulated GM images were smoothed with a Gaussian kernel of 8 mm full width at half maximum. We utilized the mean GM map (threshold = 0.2) of all the participants to obtain a group brain mask for subsequent analysis. DTI data were preprocessed using the PANDA (Pipeline for Analyzing braiN Diffusion imAges) toolbox (29). First, all DICOM files were converted into the NIfTI format. Then, a brain mask was estimated, and the non-brain spaces were removed from the raw images. After that, each diffusion-weighted image was coregistered to the b0 image using an affine transformation to correct the eddy current–induced distortions and simple head-motion artifacts with corresponding adjustments to the diffusion gradient directions. A rigorous visual inspection was performed throughout preprocessing to ensure data quality and registrations. Finally, an atlas-based approach was applied to extract regional diffusion metrics, including fractional anisotropy (FA) and mean diffusivity (MD), across 20 WM tracts. The regional FA and MD values were obtained by averaging voxel-wise measures within each ROI defined by the Johns Hopkins University white-matter tractography atlas (30).

### 2.5 Statistical analysis

Data analyses were conducted using SPSS 25.0, Mplus 8.3, and Process version 3.5. First, we explored the cognitive characteristics of three groups. After controlling for age, sex, and years of education, a series of analyses of variance (ANOVAs) was performed to compare cognitive performance between three groups. Subsequently, the *Post-hoc* pairwise t-tests were conducted when the ANOVA results were statistically significant after false discovery rate (FDR) correction for multiple comparisons.

Regarding to brain structural features, we first conducted voxel-wise comparisons of GM volume using SPM12. The threshold for the resulting statistical parametric maps was established at *p* < 0.001 (uncorrected) and then family-wise error (FWE) corrected for multiple comparisons at *p* < 0.05. Then, the DTI metrics of the atlas-based tract ROIs were analyzed using analysis of covariance (ANCOVA) in which the age, sex, and years of education were treated as covariates. Moreover, the *Post-hoc* pairwise t-tests were conducted for those significant ANCOVA results after FDR correction.

Additionally, both correlation and partial correlation analyses were performed to explore the relations between mean cortical SUVr and brain regions that showed significant differences between two MCI subgroups, with age, sex, and years of education controlled. The relations between cognitive performance and brain structural measures were evaluated in the same way. Finally, to evaluate the association between WM integrity, GM volume, and cognitive performance, we performed mediation analyses among all participants. Variables in the mediation model were defined based on the above results, and the bootstrapping method was applied to test the significance of the indirect effect of a mediator. The bootstrapped 95% confidence interval (CI) for the total indirect effect was based on 5,000 samples, and the absence of zero in the CI was considered to indicate the significance of the point estimate. Additionally, the causal steps approach was applied as a complementary analysis.

## 3 Results

### 3.1 Demographic, clinical, and neuropsychological characteristics

Demographic and cognitive data are presented in Table 1. The mean age of all participants was 68.14 years (SD = 8.225), 68.1% were female, and the average education level was 11.78 years (SD = 3.474). There were no statistically significant differences in age, sex, or education among the three groups.

**Table 1:** Demographic information and neuropsychological characteristics of three groups.

Items	NC	Aβ_neg_ MCI	Aβ_pos_ MCI	F/*χ*^2^	*p*
Gender (M/F)	9/23	7/12	6/12	0.441	0.802
Age	67.25 ± 5.92	67.68 ± 9.11	70.22 ± 10.61	0.788	0.459
Education	11.36 ± 2.90	10.95 ± 3.98	13.39 ± 3.52	2.858	0.064
General Cognitive Function					
MMSE	29.03 ± 1.56	24.84 ± 2.69	24.11 ± 2.49	38.598	< 0.001^ab^
Episodic Memory					
AVLT-delay	6.72 ± 2.74	1.11 ± 1.97	1.17 ± 1.54	44.626	< 0.001^ab^
AVLT-total	32.72 ± 9.41	14.47 ± 7.21	12.28 ± 5.75	38.535	< 0.001^ab^
ROCF-delay	16.13 ± 7.09	9.65 ± 7.04	10 ± 7.16	4.897	0.012^a^
Working Memory					
DST	13.36 ± 2.32	11.58 ± 1.92	11.17 ± 2.36	6.774	0.003^b^
DST-forward	8.19 ± 1.11	7.58 ± 1.54	7.06 ± 1.59	5.078	0.010^b^
DST-backward	5.16 ± 1.70	4 ± 1.05	4.11 ± 1.28	4.577	0.015
Spatial Processing					
ROCF-copy	35.06 ± 1.78	33.17 ± 4.93	31.83 ± 6.81	2.713	0.077
CDT	25.81 ± 4.10	22.56 ± 5.93	22.39 ± 7.16	1.845	0.169
Language					
VFT	46.97 ± 8.49	30.26 ± 10.60	28.94 ± 8.63	24.549	< 0.001^ab^
BNT	25.25 ± 1.97	20.47 ± 7.70	20.77 ± 4.28	7.885	0.001^a^
Attention					
SDMT	33 ± 10.85	22.81 ± 11.29	17 ± 8.48	8.527	< 0.001^ab^
TMT-A (s)	59.69 ± 15.43	84.47 ± 38.40	100.94 ± 41.01	10.422	< 0.001^ab^
Executive Function					
SCWT-C (s)	76.23 ± 17.40	99 ± 29.829	150.22 ± 64.60	16.735	< 0.001^bc^
TMT-B (s)	148.78 ± 47.09	221.44 ± 111.00	261.47 ± 80.6	15.107	< 0.001^ab^

*Values are the means ± standard deviations. Abbreviations: NC, normal control; Aβ_neg_ MCI, amyloid-negative MCI patients; Aβ_pos_ MCI, amyloid-positive MCI patients; MMSE, Mini-Mental State Examination; AVLT, Auditory Verbal Learning Test; DST, Digit Span Test; ROCF, Rey-Osterrieth Complex Figure; CDT, Clock-Drawing Test; VFT, Verbal Fluency Test; BNT, Boston Naming Test; SDMT, Symbol Digit Modalities Test; TMT, Trail Making Test; SCWT, Stroop Color and Word Test; s, second. ^a^*Post-hoc* paired comparisons showed significant group differences between NC and Aβ_neg_ MCI. ^b^*Post-hoc* paired comparisons showed significant group differences between NC and Aβ_pos_ MCI. ^c^*Post-hoc* paired comparisons showed significant group differences between Aβ_neg_ MCI and Aβ_pos_ MCI.

**Figure 1:**
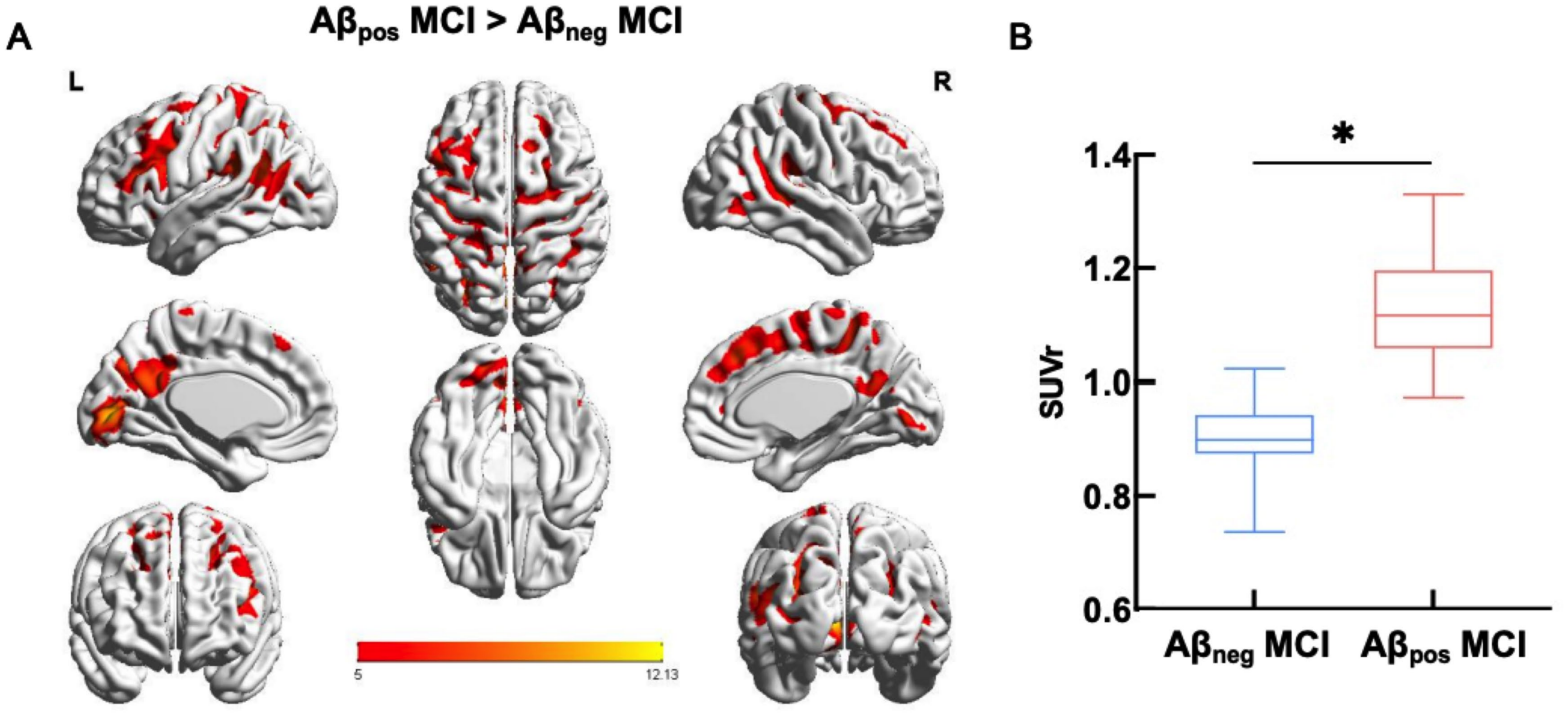
Aβ deposition of the two MCI subgroups. **(A)**. Mean between-group differences in global SUVr. **(B)** Box chart of the global SUVr. Abbreviations: Aβ_neg_ MCI, amyloid-negative MCI patients; Aβ_pos_ MCI, amyloid-positive MCI patients; SUVr, standardized uptake value ratio.

The contrast between Aβ_neg_ and Aβ_pos_ MCI of 18F-florbetapir uptake was shown in Figure 1. The quantitative analysis revealed the SUVr in Aβ_pos_ MCI group was significantly higher than in Aβ_neg_ MCI group (*t* = 7.710, *p* < 0.001), supporting the reliability of the visual read-based grouping.

For the neuropsychological tests, both Aβ_neg_ and Aβ_pos_ MCI patients performed worse than the participants in NC group. As shown in Table 1, relative to NC group, both Aβ_neg_ and Aβ_pos_ MCI showed lower scores on the tests of episodic memory, executive function, attention, and language. Additionally, Aβ_pos_ MCI patients showed a specific impairment in working memory, as reflected by lower scores in DST. In detail, both MCI subgroups showed similar declines compared with NCs in MMSE, AVLT, CVFT, SDMT, and TMT performance, while the only statistically significant difference between two MCI subgroups was observed in executive function (SCWT-C, *p* = 0.011).

### 3.2 Brain structural characterizations of MCI patients with different amyloid burden

**Figure 2:**
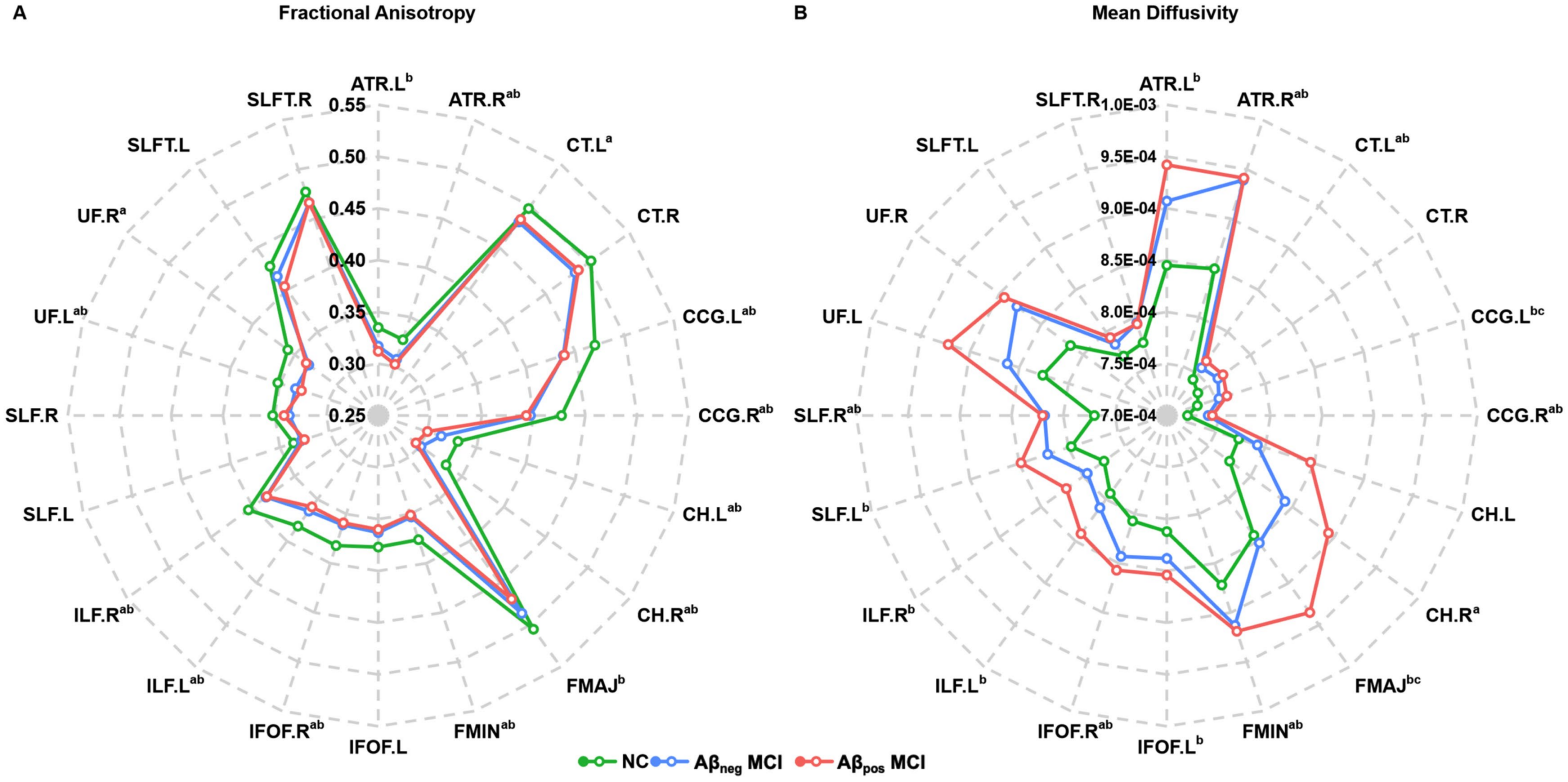
Radar plot of the differences in the atlas-based tract ROIs among the NC, Aβ_pos_ MCI and Aβ_neg_ MCI groups. **(A)** Fractional anisotropy and group differences in each atlas-based tract ROI among three groups. **(B)** Mean diffusivity and group differences in each atlas-based tract ROI among three groups. Statistically significant differences after Bonferroni correction are marked with superscripts. ^a^*Post-hoc* paired comparisons showed significant group differences between NC and Aβ_neg_ MCI. ^b^*Post-hoc* paired comparisons showed significant group differences between NC and Aβ_pos_ MCI. ^c^*Post-hoc* paired comparisons showed significant group differences between Aβ_neg_ MCI and Aβ_pos_ MCI. Abbreviations: NC, normal control; Aβ_neg_ MCI, amyloid-negative MCI patients; Aβ_pos_ MCI, amyloid-positive MCI patients. Abbreviations of white matter tracts are listed in the Supplementary Table S1.

Atlas-based analysis of WM tract integrity is presented in Figure 2 (For the abbreviations of WM tracts, see Supplementary Table S1). Overall, a graded pattern of WM disruption was observed, with the Aβ_neg_ MCI group showing mild alterations, and the Aβ_pos_ MCI displaying more pronounced abnormalities. Figure 2A presents the comparison of FA among three groups, where 14 of the 20 atlas-based WM tract ROIs exhibit significant intergroup differences. Specifically, *post-hoc* analyses found that both Aβ_neg_ MCI and Aβ_pos_ MCI patients showed decreased FA in the bilateral cingulum cingulate gyrus part (CCG), cingulum hippocampus part (CH), inferior longitudinal fasciculus (ILF), right anterior thalamic radiation (ATR), inferior fronto-occipital fasciculus (IFOF), left uncinate fasciculus (UF), and forceps minor (FMIN). In addition, Aβ_neg_ MCI showed lower FA in the left corticospinal tract (CT) and right UF, while Aβ_pos_ MCI show lower FA in the left ATR and the forceps major (FMAJ), with no significant inter-group differences found. Differences in MD between groups were displayed in Figure 2B. Similarly, the two MCI subgroups showed extensive increased MD in 16 of the 20 WM tract ROIs. In addition, when comparing to Aβ_neg_ MCI patients, Aβ_pos_ MCI patients showed significantly greater MD in the left CH and FMAJ (details provided in Supplementary Table S2).

**Figure 3:**
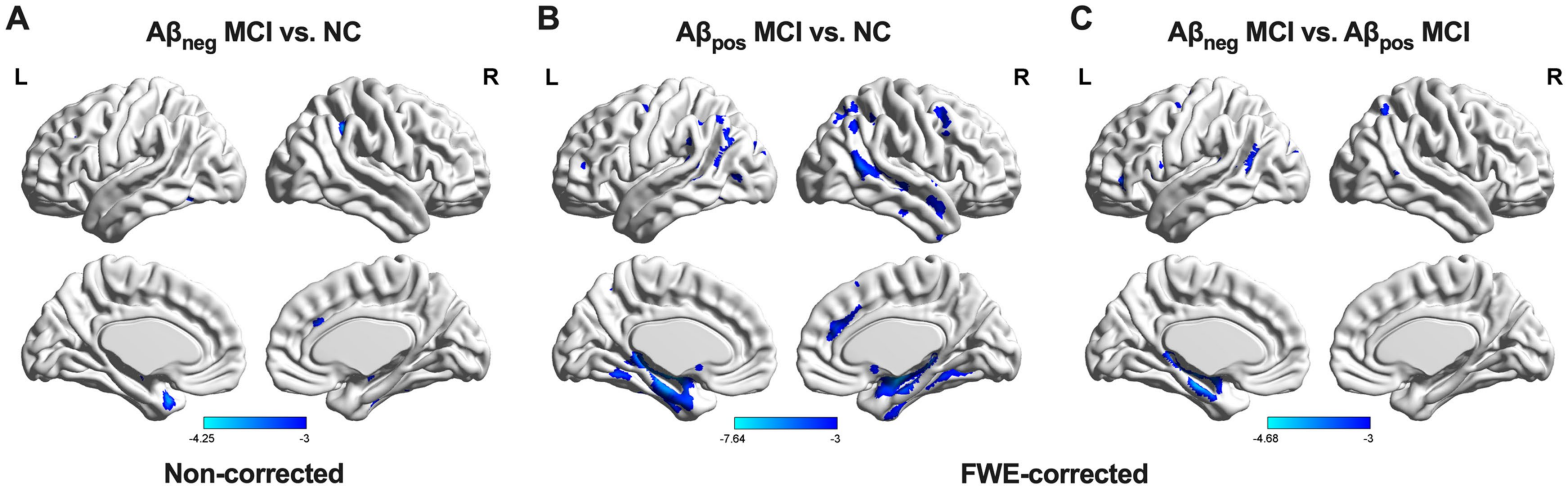
GM volume differences among the three groups. **(A)** Group differences between NC and Aβ_neg_ MCI. **(B)** Group differences between NC and Aβ_pos_ MCI. **(C)** Group difference between Aβ_neg_ MCI and Aβ_pos_ MCI. Abbreviations: NC, normal control; Aβ_neg_ MCI, amyloid-negative MCI patients; Aβ_pos_ MCI, amyloid-positive MCI patients; L, left; R, right.

Contrast results of GM volume were presented in Figure 3. Relative to NC, Aβ_pos_ MCI showed significant atrophy in the bilateral hippocampus (HIP) and left parahippocampus. In the Aβ_neg_ MCI group, an atrophy trend was observed in the right supramarginal gyrus, inferior temporal gyri, left temporal pole, and fusiform gyrus; however, these changes did not reach statistical significance. Relative to Aβ_neg_ MCI, Aβ_pos_ MCI exhibited more atrophy in the left HIP.

In summary, both MCI subgroups showed widespread WM tract disruptions, whereas significant GM atrophy was observed only in the Aβ_pos_ MCI group. Moreover, the structural differences between the two MCI subgroups were mainly located in the left hippocampal region. Therefore, MD of the left CH and FMAJ, together with GM volume of the left HIP, was selected as main focus in later analyses.

### 3.3 Relations between SUVr, gray matter volume and white matter integrity

**Figure 4:**
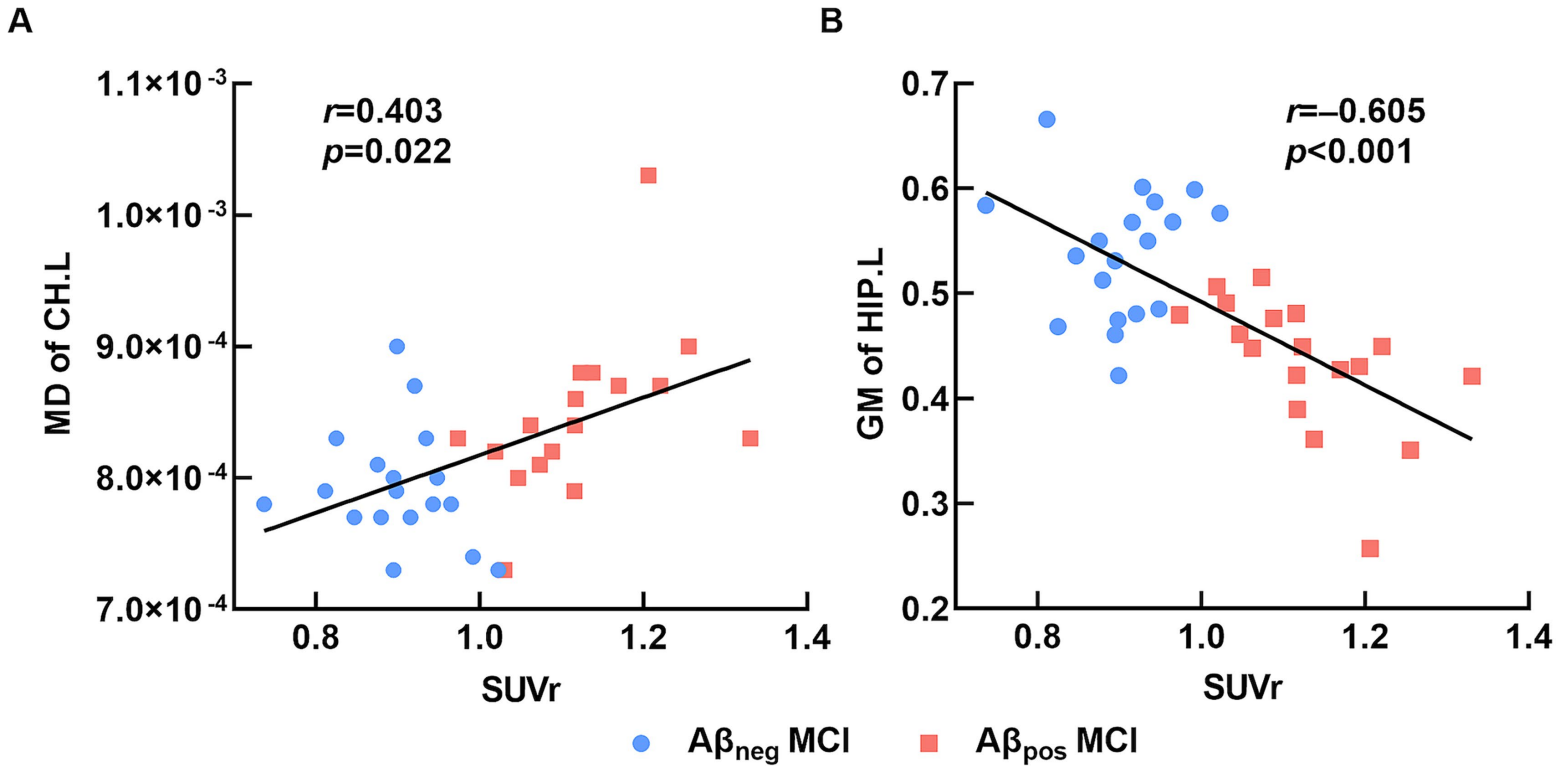
Relation between brain structure and SUVr. **(A)** The average mean cortical SUVr was significantly positively correlated with MD of the left cingulum (hippocampus). **(B)** The average mean cortical SUVr was significantly negatively correlated with GM volume of the left hippocampus. Abbreviations: MD, mean diffusivity; CH.L, left of cingulum (hippocampus); GM, gray matter; HIP.L, left hippocampus; Aβ_neg_ MCI, amyloid-negative MCI patients; Aβ_pos_ MCI, amyloid-positive MCI patients; SUVr, standardized uptake value ratio.

To further investigate the influence of amyloid burden on brain structure, we calculated the correlations between mean cortical SUVr and brain regions that showed significant intergroup differences among all MCI participants. Results showed that the mean cortical SUVr was positively associated with MD of the left CH (*r* = 0.508, *p* = 0.002), negatively associated with GM volume of the left HIP (*r* = −0.677, *p* < 0.001), and not significantly associated with MD of FMAJ (*r* = 0.191, *p* = 0.273). After controlling for age, sex, and years of education, the SUVr remained significantly correlated with GM of the left HIP (*r* = −0.605, *p* = 0.001) and MD of the left CH (*r* = 0.403, *p* = 0.022; Figure 4). In conclusion, the Aβ deposition may lead to severe disruptions of WM integrity and GM atrophy in the left hippocampal region among MCI patients.

### 3.4 Associations between brain structure characterizations and cognitive performance

This study also explored the relations between cognitive performance and brain structure characterizations among all MCI patients. After adjusting for age, sex, and years of education, we found MD of left CH was negatively correlated with AVLT-delay (*r* = −0.489, *p* = 0.034) while the correlation between cognitive performance and MD of FMAJ and GM of the left HIP were not significant. Therefore, the AVLT-delay was selected for further mediation analysis.

**Figure 5:**
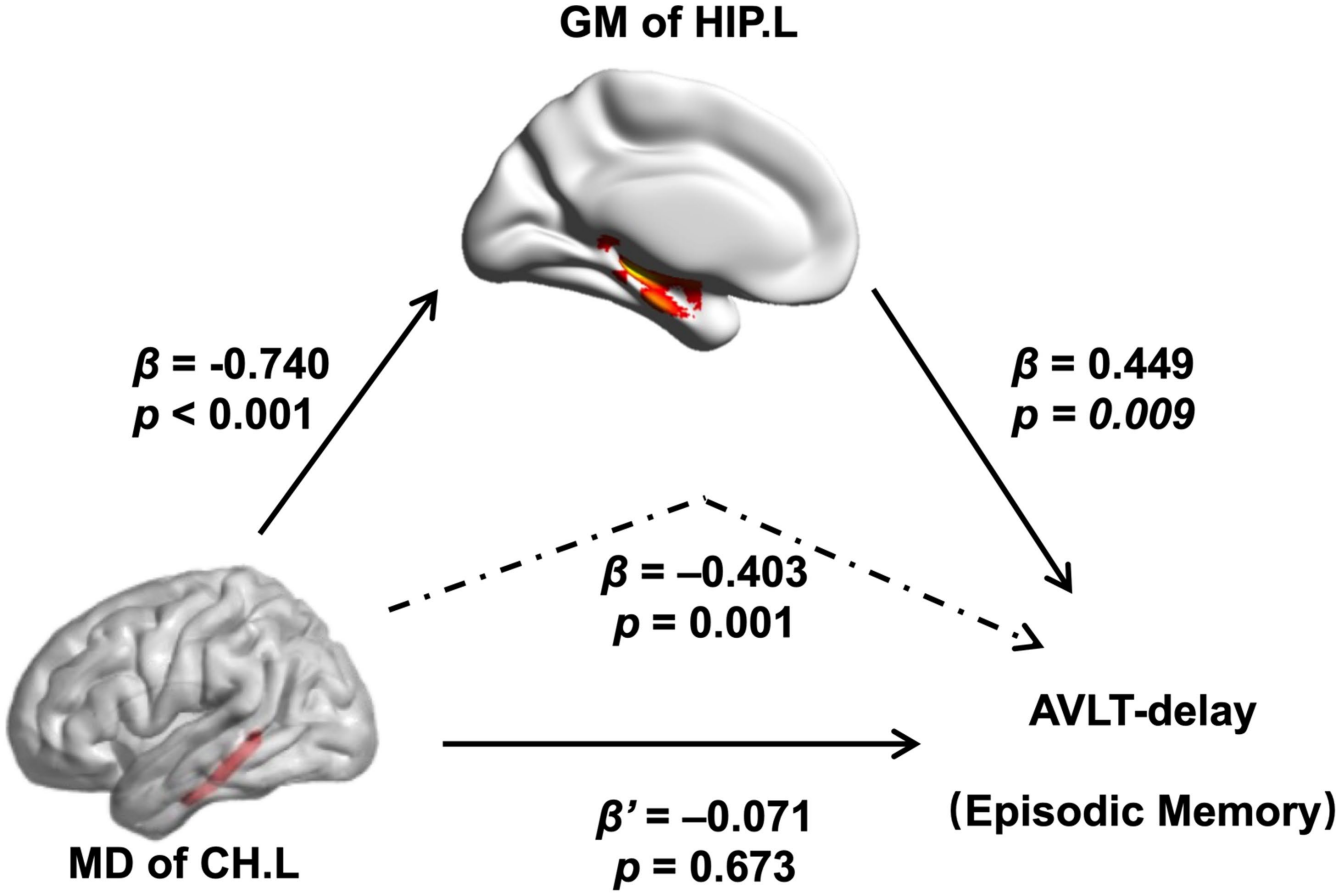
Relations of MD of the left CH with both GM volume of the left hippocampal and performance of the AVLT-delay. As indicated by the regression coefficients and the *p* values, there was a significant mediating effect. Abbreviations: MD, mean diffusivity; CH.L, left of cingulum (hippocampus); GM, gray matter; HIP.L, left hippocampus; AVLT, auditory verbal learning test.

In this study, we further assessed the associations between altered brain structures and cognitive performance. In the mediation model, the independent factor was the MD of the left CH, and the dependent variable was the AVLT-delay score, which represents episodic memory performance, and the proposed mediator was the GM of the left HIP. As shown in Figure 5, the GM volume of the left HIP mediated the effect of the MD of the left CH on the AVLT-delay score. The bootstrap method revealed that the standardized indirect effect value in this mediation model (−0.333: 95% CI, −0.590 to –0.115) did not include zero, confirming a significant indirect effect. Furthermore, the mediation effect was not significant when the GM volume of the left HIP was the independent factor, and the MD of the left CH was proposed to be the mediator (details are provided in the Supplementary material).

## 4 Discussion

Herein, this study explored the cognitive and neural characteristics of Aβ_neg_ MCI patients, focusing on their similarities and differences with participants in Aβ_pos_ MCI group. Our results showed significant multidomain cognitive decline and extensive disruptions of WM integrity in both Aβ_neg_ and Aβ_pos_ MCI subgroups, with significant hippocampal atrophy observed only in Aβ_pos_ MCI group. In addition, the major differences between two MCI subgroups were found in the left hippocampal region, which also showed a close relation with episodic memory dysfunction. Our findings suggested hippocampal damage in Aβ_neg_ MCI patients may contribute to advanced cognitive decline and dementia progression, requiring more attention.

Previous studies have explored cognitive differences between MCI patients with and without Aβ deposition, yielding inconsistent results. Some reported that Aβ_pos_ MCI patients exhibited prominent episodic memory impairments, while Aβ_neg_ patients showed deficits in working memory and executive function (31, 32). In contrast, Mendes et al. (33) found no significant cognitive differences between Aβ_neg_ and Aβ_pos_ MCI groups. These discrepancies may arise from variations in diagnostic criteria and cognitive assessment tools for MCI. The current study explored cognitive differences by recruiting participants in the same project and conducting uniform neuropsychological tests, thereby enhancing the reliability of our findings. Compared with NCs, both Aβ_pos_ MCI and Aβ_neg_ MCI patients exhibited extensive cognitive deficits. Specifically, similar patterns of impairment were found in the domains of episodic memory and processing speed, which are closely linked to the progression of dementia. Disruption of episodic memory is widely regarded as the most pronounced cognitive defect in AD. Stable preclinical episodic memory deficits can be detected 6.6–7.3 years before the diagnosis of AD (34). Additionally, attention dysfunction is also commonly reported (35) and may aid in the clinical differentiation of AD from other types of dementia (36). Our results suggest that a portion of individuals with Aβ_neg_ MCI may be in a very early stage of AD pathology. Given this possibility, follow-up visits could be considered for Aβ_neg_ MCI patients, especially those showing similarities to Aβ_pos_ MCI patients in AD-related cognitive deficits.

This study found significant WM damage in both Aβ_pos_ and Aβ_neg_ MCI groups, while notable GM atrophy was only observed in the Aβ_pos_ MCI group. Previous studies have reported that progressive WM degeneration and demyelination are important early pathological features of AD (37) and can be detected 10 years before the onset of clinical symptoms (38). In addition, an increasing number of studies have reported that WM alterations may appear prior to GM changes such as hippocampus atrophy (38, 39). Taken together, these findings may explain why WM damage is more pronounced than the GM damage. Consistent with our finding, a recent study found that MCI patients with negative Aβ deposition also exhibited widespread WM structural disruptions. Besides, that study also found that WM microstructural damage in the corpus callosum and posterior cingulate cortex was closely associated with an abnormal plasma Aβ42/40 ratio (40). Overall, these findings suggested that WM changes in specific regions, such as the temporal lobe, may be linked to neurodegenerative processes associated with AD pathology. Further longitudinal research is needed to clarify the pathological relationship between WM microstructural changes occurring before significant Aβ deposition and subsequent disease progression and cognitive decline.

Our analysis also revealed that the differences in MD between the two MCI subgroups were located at the left CH and FMAJ. Posterior brain regions have been reported to be most affected by AD pathology in DTI studies (41). A decrease in WM integrity in the posterior cingulate and parahippocampus has been reported in both AD and MCI patients (41, 42). The corpus callosum is the largest association fiber connecting the bilateral hemispheres. Atrophy and reduced integrity of the corpus callosum have been found in AD pathology (43). As a posterior projection of the splenium of the corpus callosum, the FMAJ shows increased MD even 5–10 years before the patient becomes symptomatic (38). In conclusion, these results suggested that Aβ deposition may specifically cause additional impairments in AD-related WM fibers in the context of a similar pattern of WM degeneration in MCI patients.

Furthermore, we noticed that MD exhibited more pronounced group differences than FA in this study. This discrepancy may be attributed to the distinct characteristics of these metrics (42). Aβ deposition can lead to axonal injury and demyelination, as indicated by a transverse diffusion increase in the DTI index. However, this process could be masked in the reorganization of fiber tracts and reactivity of glial (44). MD is more sensitive to the loss of anisotropy than FA does, the reason could be due to calculation as it is derived from 3 eigenvectors of the diffusion tensor, but FA is derived from the normalized variance of these eigenvectors, reflecting the degree of directionality of water diffusion. Therefore, it is understandable to find more differences in MD than in FA (45).

In this study, we observed a significant correlation between Aβ deposition and increased MD in the left CH as well as atrophy of the left HIP; however, few correlations with cognitive performance were found. These discrepancies may be explained by the characteristics of Aβ pathology progression. The Aβ accumulation starts 10 to 20 years before an AD diagnosis and could cause damage to oligodendrocytes, WMLs (46) and GM atrophy (47). Prior studies reported that Aβ reaches a steady level before cognitive decline occurs (48). Accordingly, Aβ deposition may not synchronize with the clinical outcome of cognitive impairment, despite its effect on brain structure and function (49, 50). Building upon these results, we further explored the relations between changes in brain structure and cognitive deficits. We found that the effect of WM disruption on episodic memory was mediated by GM volume while the mediation effect was not significant when the WM disruption were treated as mediators. Numerous studies have reported that WMLs are not only secondary to GM damage (51) but can also be directly affected by AD independent of GM degeneration (52). In addition, WM degeneration could lead to neighboring GM structure damage in AD patients (53). For example, disruption of the cingulate fasciculus could cause hippocampal atrophy in mild AD patients and further lead to episodic memory deficits (54). Taken together, our findings suggest that WMLs, especially those involving the hippocampal region, may serve as potential markers for identifying individuals at high risk of AD in the very early preclinical stage.

By including an NC group, we were able to delineate the distinct cognitive and neural characteristics of Aβ_neg_ and Aβ_pos_ MCI groups. We observed that participants in both MCI subgroups exhibited a similar degree of cognitive impairment and widespread WM disruptions. Moreover, across all MCI participants, Aβ deposition was associated with additional AD-related GM atrophy within a broadly similar neurodegenerative pattern. Notably, WM damage in the hippocampal regions may contribute to episodic memory deficits by exacerbating GM deterioration. Taken together, our findings suggest that even in the absence of significant Aβ accumulation, some MCI individuals show cognitive and neural alterations resembling those observed in Aβ_pos_ MCI. This suggests that pathological changes related to AD may already be underway before the onset of detectable amyloid positivity. Therefore, Aβ deposition alone may a insufficient for predicting AD progression (55). Continued attention to Aβ_neg_ MCI—particularly through integrating other biomarkers—remains essential.

Several limitations of this study should be noted. First, this study did not collect additional AD-related biomarkers beyond Aβ (e.g., tau PET or the APOE ε4 allele). This absence may have limited the accuracy in characterizing the pathological status of the MCI participants, particularly those in the Aβ_neg_ MCI group. Future studies incorporating more biomarkers are needed to better capture the biological heterogeneity of Aβ_neg_ MCI and to identify individuals at greater risk for advanced cognitive decline and AD progression. Second, this study adopted a cross-sectional design, which limited the examination of how Aβ_neg_ MCI progress over time. Longitudinal follow-up is required to determine which features may correlat with a higher risk of subsequent disease progression in Aβ_neg_ MCI. Such investigations would facilitate the identification and characterization of Aβ_neg_ MCI patients who may be at a very early stage of AD, prior to the accumulation of amyloid reaching the positivity threshold. Lastly, not all NC participants underwent PET scan, which may have partially influenced the characterization of Aβ_pos_ and Aβ_neg_ MCI subgroups. Our supplementary analysis showed that NC participants without PET data demonstrated better cognitive performance than those with PET scans (see Supplementary Table S3), suggesting that the control standard may not become more lenient by the potential inclusion of Aβ_pos_ NC individuals. Future studies should refine the recruitment criteria for NC participants to enhance the statistical validity and robustness of group comparisons.

As of today, there is no curable treatment for dementia. Therefore, expanding the intervention window for individuals at high risk of AD remains one of the most critical preventive strategies. In this study, our findings suggested that Aβ_neg_ MCI participants with episodic memory and processing speed impairments, accompanied by hippocampal changes may have a higher risk of developing AD. These findings may help identify individuals at high risk of disease conversion and enhance the understanding of the pathophysiological changes underlying the prodromal stage of AD.

## Data Availability

The raw data supporting the conclusions of this article will be made available by the authors, without undue reservation.

## Footnotes

1 https://www.fil.ion.ucl.ac.uk/spm12.
