# Supplementary material for: Neuropsychological and neuroimaging characteristics of patients with mild cognitive impairment and negative-amyloid deposition
Source: Front Neurol. 2026 Jan 5;16:1658712. doi: 10.3389/fneur.2025.1658712 (PMC12812577; doi:10.3389/fneur.2025.1658712)
Supplement: Supplementary file 1 [file Table_1.docx]

Supplementary Material

| **index** | **Region** | **abbr.** |
| --- | --- | --- |
| 1 | Anterior.thalamic.radiation.L | ATR.L |
| 2 | Anterior.thalamic.radiation.R | ATR.R |
| 3 | Corticospinal.tract.L | CT.L |
| 4 | Corticospinal.tract.R | CT.R |
| 5 | Cingulum.(cingulate.gyrus).L | CCG.L |
| 6 | Cingulum.(cingulate.gyrus).R | CCG.R |
| 7 | Cingulum.(hippocampus).L | CH.L |
| 8 | Cingulum.(hippocampus).R | CH.R |
| 9 | Forceps.major | FMAJ |
| 10 | Forceps.minor | FMIN |
| 11 | Inferior.fronto-occipital.fasciculus.L | IFOF.L |
| 12 | Inferior.fronto-occipital.fasciculus.R | IFOF.R |
| 13 | Inferior.longitudinal.fasciculus.L | ILF.L |
| 14 | Inferior.longitudinal.fasciculus.R | IL.R |
| 15 | Superior.longitudinal.fasciculus.L | SLF.L |
| 16 | Superior.longitudinal.fasciculus.R | SLF.R |
| 17 | Uncinate.fasciculus.L | UF.L |
| 18 | Uncinate.fasciculus.R | UF.R |
| 19 | Superior.longitudinal.fasciculus.(temporal.part).L | SLFT.L |
| 20 | Superior.longitudinal.fasciculus.(temporal.part).R | SLFT.R |

**Table S1.** Abbreviations of the white matter tracts of the ROIs.

**Table S2.** Diffusion Coefficient Differences of Atlas-based Tract ROIs among the Three Groups.

|  | **FA** | | | | | **MD(10^-4^)** | | | | |
| --- | --- | --- | --- | --- | --- | --- | --- | --- | --- | --- |
| **Tracts**^#^ | **NC** | **Aβ_neg_ MCI** | **Aβ_pos_ MCI** | ***F*** | ***p*** | **NC** | **Aβ_neg_ MCI** | **Aβ_pos_ MCI** | ***F*** | ***p*** |
| ATR.L | 0.335 0.017 | 0.317 0.028 | 0.312 0.027 | 4.974 | **0.010^b^** | 8.45 0.763 | 9.07 1.01 | 9.42 1.19 | 5.163 | **0.009^b^** |
| ATR.R | 0.327 0.015 | 0.307 0.030 | 0.302 0.025 | 6.158 | **0.004^ab^** | 8.49 0.665 | 9.39 1.32 | 9.41 1.10 | 5.897 | **0.005^ab^** |
| CCG.L | 0.470 0.029 | 0.438 0.030 | 0.439 0.030 | 7.294 | **0.002^ab^** | 7.31 0.249 | 7.53 0.293 | 7.61 0.318 | 5.597 | **0.006^ab^** |
| CCG.R | 0.427 0.031 | 0.397 0.038 | 0.393 0.039 | 6.212 | **0.004^ab^** | 7.20 0.228 | 7.40 0.373 | 7.44 0.339 | 3.264 | 0.046 |
| CH.L | 0.331 0.022 | 0.314 0.024 | 0.300 0.023 | 8.995 | **0.000^ab^** | 7.73 0.458 | 7.92 0.443 | 8.46 0.616 | 9.829 | **0.000^bc^** |
| CH.R | 0.331 0.027 | 0.301 0.033 | 0.295 0.026 | 9.629 | **0.000^ab^** | 7.75 0.529 | 8.41 0.926 | 8.93 0.867 | 11.804 | **0.000^ab^** |
| CT.L | 0.497 0.017 | 0.481 0.019 | 0.484 0.023 | 3.816 | **0.028^a^** | 7.43 0.194 | 7.57 0.275 | 7.65 0.407 | 2.534 | 0.088 |
| CT.R | 0.504 0.018 | 0.485 0.035 | 0.489 0.021 | 2.926 | 0.062 | 7.37 0.173 | 7.61 0.456 | 7.67 0.373 | 4.424 | **0.016^a^** |
| FMAJ | 0.505 0.020 | 0.486 0.037 | 0.469 0.042 | 5.473 | **0.007^b^** | 8.43 0.684 | 8.52 0.505 | 9.35 1.06 | 5.993 | **0.004^bc^** |
| FMIN | 0.376 0.017 | 0.353 0.028 | 0.351 0.023 | 9.142 | **0.000^ab^** | 8.72 0.351 | 9.13 0.537 | 9.19 0.452 | 8.058 | **0.001^ab^** |
| IFOF.L | 0.377 0.019 | 0.363 0.026 | 0.360 0.024 | 3.043 | 0.056 | 8.12 0.275 | 8.38 0.452 | 8.54 0.454 | 5.381 | **0.007^b^** |
| IFOF.R | 0.382 0.019 | 0.361 0.029 | 0.359 0.025 | 5.370 | **0.007^ab^** | 8.07 0.264 | 8.43 0.520 | 8.57 0.482 | 8.049 | **0.001^ab^** |
| ILF.L | 0.382 0.019 | 0.364 0.023 | 0.359 0.023 | 5.797 | **0.005^ab^** | 7.93 0.259 | 8.10 0.250 | 8.41 0.662 | 5.129 | **0.009^b^** |
| ILF.R | 0.405 0.020 | 0.384 0.026 | 0.383 0.021 | 6.015 | **0.004^ab^** | 7.75 0.280 | 7.95 0.319 | 8.20 0.448 | 6.769 | **0.002^b^** |
| SLF.L | 0.336 0.017 | 0.328 0.017 | 0.325 0.019 | 1.892 | 0.16 | 7.97 0.328 | 8.21 0.466 | 8.48 0.420 | 7.313 | **0.002^b^** |
| SLF.R | 0.352 0.019 | 0.336 0.029 | 0.341 0.019 | 2.390 | 0.101 | 7.70 0.333 | 8.18 0.728 | 8.20 0.424 | 7.438 | **0.001^ab^** |
| SLFT.L | 0.428 0.035 | 0.416 0.034 | 0.404 0.026 | 2.369 | 0.103 | 7.71 0.348 | 7.85 0.335 | 7.93 0.349 | 1.693 | 0.193 |
| SLFT.R | 0.477 0.039 | 0.466 0.045 | 0.466 0.031 | 0.375 | 0.689 | 7.74 0.494 | 7.94 0.527 | 7.93 0.266 | 1.094 | 0.342 |
| UF.L | 0.352 0.016 | 0.334 0.019 | 0.328 0.028 | 6.000 | **0.004^ab^** | 8.26 0.336 | 8.62 0.431 | 9.22 1.39 | 5.679 | **0.006** |
| UF.R | 0.358 0.021 | 0.333 0.037 | 0.336 0.025 | 4.999 | **0.010^a^** | 8.15 0.351 | 8.79 1.46 | 8.94 0.901 | 4.018 | **0.023** |

Values are the means and standard deviations. FA: fractional anisotropy; MD: mean diffusivity; NC: normal control; Aβ_neg_ MCI: amyloid-negative MCI patients; Aβ_pos_ MCI: amyloid-positive MCI patients.

The *p* values shown in bold indicate p < 0.05 after FDR correction.

^a^*Post-hoc* paired comparisons showed significant group differences between NC and Aβ_neg_ MCI.

^b^*Post-hoc* paired comparisons showed significant group differences between NC and Aβ_pos_ MCI.

^c^*Post-hoc* paired comparisons showed significant group differences between Aβ_neg_MCI and Aβ_pos_ MCI.

^#^ For the abbreviations of WM tracts, see Supplementary Table S1.

**Table S3.** Neuropsychological Characteristics for NC participants with and without PET scan.

|  | **NC without PET**  **n = 22** | **NC with PET**  **n = 10** | **t** | ***p*** |
| --- | --- | --- | --- | --- |
| MMSE | 27.60±1.78 | 29.68±0.89 | -3.51 | 0.005^*^ |
| AVLT-total | 22.30±3.97 | 37.45±6.98 | -6.37 | < 0.001^*^ |
| AVLT-delay | 3.80±1.62 | 8.05±2.01 | -5.85 | < 0.001^*^ |
| ROCF-delay | 15.80±6.05 | 16.27±7.65 | -0.17 | 0.865 |
| DST | 12.00±2.29 | 13.91±2.14 | -2.21 | 0.035^*^ |
| DST-forward | 8.00±1.58 | 8.27±0.88 | -0.49 | 0.636 |
| DST-backward | 4.00±1.32 | 5.64±1.62 | -2.68 | 0.012^*^ |
| ROCF-copy | 34.60±2.50 | 35.27±1.35 | -0.99 | 0.329 |
| CDT | 25.22±5.61 | 26.05±3.44 | -0.50 | 0.620 |
| VFT | 42.00±7.96 | 49.23±7.89 | -2.40 | 0.023^*^ |
| BNT | 24.70±1.83 | 25.50±2.02 | -1.07 | 0.294 |
| SDMT | 26.20±9.27 | 36.09±10.24 | -2.60 | 0.014^*^ |
| TMT-A (s) | 69.90±17.63 | 55.05±12.09 | 2.78 | 0.009^*^ |
| SCWT-C (s) | 82.10±16.69 | 73.43±17.42 | 1.31 | 0.200 |
| TMT-B (s) | 174.80±45.04 | 136.95±43.98 | 2.24 | 0.033^*^ |

Abbreviations: NC: normal control; MMSE: Mini-Mental State Examination; AVLT: Auditory Verbal Learning Test; DST: Digit Span Test; ROCF: Rey-Osterrieth Complex Figure; CDT: Clock-Drawing Test; VFT: Verbal Fluency Test; BNT: Boston Naming Test; SDMT: Symbol Digit Modalities Test; TMT: Trail Making Test; SCWT: Stroop Color and Word Test.

^*^ Values are the means ± standard deviations.*p* < 0.05 was considered significant.

# *Mediation Analysis*

Another exploratory mediation analysis was performed. In this mediation analysis, the independent factor was the GM of HIP.L and dependent variable was AVLT-delay which represented the episodic memory performances. The mediator was the MD of the CH.L.

The bootstrapping method was used to test the significance of the indirect effect of a mediator. The bootstrap 95% confidence interval (CI) for the total indirect effect was based on 5000 samples. The absence of zero in the CI was considered to indicate the significance of the point estimate (p < 0.05). The standardized indirect effect value in this model (-0.053: 95% CI, -0.219 – 0.276) did include zero, confirming that indirect effect is not significant.
